# “Look Versus See”: Does Varying Fellow Eye Contrast Affect Perception of the Amblyopic Eye?

**DOI:** 10.1167/iovs.67.4.6

**Published:** 2026-04-03

**Authors:** Ibrahim M. Quagraine, Shi Shi, Gokce Busra Cakir, Jordan Murray, Aasef G. Shaikh, Fatema F. Ghasia

**Affiliations:** 1 Department of Biomedical Engineering, Case Western Reserve University, Cleveland, Ohio, United States; 2 Case Western Reserve University School of Medicine, Cleveland, Ohio, United States; 3 Cole Eye Institute, Cleveland Clinic, Cleveland, Ohio, United States; 4 Daroff-Dell'Osso Ocular Motility Laboratory, Louis Stokes Cleveland VA Medical Center, Cleveland, Ohio, United States

**Keywords:** visual search, visual attention, binocular vision, amblyopia, dichoptic therapy

## Abstract

**Purpose:**

This study aimed to evaluate how modulating fellow eye (FE) contrast during dichoptic visual search impacts amblyopic eye (AE) perception and oculomotor behavior in amblyopia. By leveraging eye-tracking data, we investigated how FE contrast and sensory–motor factors influence perceptual outcomes, distinguishing successful attentional allocation and target identification (“look and see”) from “look but failed to see” (LBFTS) and “no look, no see” errors.

**Methods:**

All participants performed visual search under monocular and dichoptic viewing with varied FE contrasts. High-resolution eye-tracking captured microsaccades (<1°), saccades (>1°), drift velocity, eye deviation via the density-based spatial clustering of applications with noise algorithm (DBSCAN) and attention patterns (heatmaps, scan paths). Search efficiency was measured as the time from image onset to the final fixation within the region of interest (look time), and perceptual accuracy was assessed via mouse-click localization of the target.

**Results:**

In amblyopic participants, full-contrast FE dichoptic viewing impaired AE target detection and prolonged look time. Reducing FE contrast improved AE performance—especially in those with mild suppression and good stereopsis—by shortening look time and reducing LBFTS errors. However, excessive FE contrast reduction (≤25%) impaired FE target detection in controls and amblyopia participants. The “look and see” trials showed higher microsaccade frequency, whereas LBFTS and “no look, no see” (i.e., incorrect) trials were linked to increased saccades, eye deviation, and increased FE drift.

**Conclusions:**

Contrast rebalancing can enhance AE attentional and perceptual performance, but optimal FE contrast varies with individual sensory–motor profiles. Our findings underscore the value of integrating visual search paradigms with eye tracking to personalize dichoptic therapies and objectively monitor treatment efficacy.

Amblyopia, commonly known as “lazy eye,” is a neurodevelopmental disorder that affects 2% to 5% of the population.^1^ It arises from early visual deprivation, anisometropia, or strabismus, or a combination thereof.^1^^,^^2^ It is increasingly recognized not merely as a monocular deficit but as a binocular vision disorder with functional deficits in both the amblyopic eye (AE) and to lesser extent the fellow eye (FE).^3^^–^^8^ Recent evidence indicates that suppression, the cortical inhibition of the perception of AE during binocular viewing, plays a central role in the pathogenesis of amblyopia.^9^^–^^11^ Suppression, along with reduced stereopsis, diminished binocular visual acuity, and impairments in oculomotor control under natural binocular viewing collectively undermine visual processing efficiency in everyday tasks in amblyopia.^3^^–^^5^^,^^8^^,^^12^^–^^16^ Traditional treatments such as patching or atropine often leave residual deficits.^7^^,^^17^^–^^23^ Emerging dichoptic therapies that present distinct stimuli to each eye aim to counteract suppression by reducing contrast of the FE stimulus to promote binocular integration.^24^^–^^28^ However, clinical outcomes remain inconsistent, particularly in pediatric populations with anisometropic or small-angle strabismic amblyopia,^19^^,^^27^^,^^29^^–^^31^ highlighting the need to better understand the sensory and motor factors that influence AE perception and affect treatment efficacy.

Visual search tasks, which require locating a target within complex visual scene, offer a robust framework to probe these factors, as they engage both sensory (perception) and motor (eye movement) processes under controlled conditions. Amblyopic individuals consistently show slower reaction times and reduced accuracy, even under binocular and monocular viewing.^32^^–^^35^ These deficits are compounded by abnormal oculomotor patterns, including deficient microsaccadic and saccadic activity and increased intersaccadic drifts, suggesting that both visual sensory and motor abnormalities contribute to the observed deficits.^32^^,^^33^^,^^36^

In our previous work, which primarily examined behavioral differences, we found that reducing FE contrast during dichoptic visual search (DiVS) significantly improved target detection. The improvements were more pronounced in individuals with anisometropic amblyopia compared to those with strabismic amblyopia,^34^ possibly because the presence or variability of eye deviation in strabismic individuals may disrupt the ability of the AE to effectively coordinate gaze toward the region of interest (ROI). Alternatively, amblyopia may impair higher-order visual processes, such as visual attention, which are essential for efficient search behavior.^37^^,^^38^ However, the contribution of these higher-order mechanisms—particularly the distinction between “looking” (fixation) and “seeing” (perception)—remains underexplored, especially in the context of perceptual failures such as “look but failed to see” (LBFTS) errors. These errors, where a stimulus is fixated but not consciously perceived,^39^^,^^40^ may result from incomplete processing during fixation or premature disengagement of attention. Such phenomena are closely linked to the brain's generative model of the visual world and the asynchronous nature of action and perception, making them especially relevant in amblyopia, where both sensory and motor deficits are compromised.^39^^–^^42^

In this study, we leveraged high-resolution eye-tracking to investigate how suppression, visual acuity deficits, stereopsis, and oculomotor dynamics contribute to perceptual failures during DiVS. Eye-tracking allows us to distinguish between different failure modes: isolated attentional allocation without perceptual recognition (“look but fail to see”) versus absence of attention allocation and subsequent perceptual failure. This differentiation offers critical insight into the mechanisms underlying perceptual deficits in amblyopia. Eye movement markers such as microsaccades (<1°) and drift velocities, which are tightly linked to perception in neurotypical individuals and known to be abnormal in amblyopia, offer a unique window into the pathophysiology of the disorder.^43^^–^^46^ Moreover, reports of new-onset or worsening strabismus in 8% to 10% of patients undergoing amblyopia treatment—including dichoptic therapy—highlight the importance of monitoring eye deviation during exposure to dichoptic stimuli.^19^^,^^31^^,^^47^^–^^49^ This study aimed to (1) quantify visual search efficiency and perception, defined as the time taken from start of the trial to last fixation within a ROI (look time); (2) measure action through the time from last fixation within a ROI to mouse-click localization of the target (action time); (3) determine whether fixations within the ROI lead to accurate target identification (“looking” vs. “seeing”); and (4) identify the sensory and motor factors underlying failures to fixate or detect the target.

We hypothesized that DiVS performance, along with eye-tracking, would serve as a novel and sensitive objective marker for evaluating an individual's ability to overcome suppression in amblyopia. By capturing both sensory and motor components of visual behavior, this approach enables precise quantification of attention allocation and perceptual success under varying FE contrast conditions. We anticipate that reducing FE contrast will facilitate binocular integration in a subset of participants, leading to improved search efficiency through the AE and concurrent enhancement of both attentional engagement and perceptual recognition, characterized as “look and see” behavior. We predicted that improvements in “look and see” behavior would correlate with clinical and sensory metrics—including depth of suppression, visual acuity, and stereopsis deficits—as well as oculomotor indicators such as reduced eye deviation and increased microsaccadic frequency.

## Methods

The study protocols were approved by The Cleveland Clinic Institutional Review Board. Written informed consent was obtained from each participant or parent/legal guardian, as mandated by the tenets of the Declaration of Helsinki. A total of 37 subjects were recruited (14 healthy controls and 23 amblyopia subjects). All participants underwent a comprehensive ophthalmic evaluation, and relevant clinical parameters were collected (Table). Amblyopia type (12 anisometropic, 11 strabismic/mixed) and severity (mild/treated, *n* = 8; moderate, *n* = 15) were classified according to the Pediatric Eye Disease Investigator Group criteria.^50^ For the purposes of this study, individuals with current or previously treated amblyopia were collectively referred to as amblyopic participants. For all measurements, participants wore optical corrections based on their cycloplegic refraction, with hyperopic adjustments made symmetrically for both eyes as clinically necessary to achieve the best-corrected visual acuity.

**Table.** Demographic and Visual Function Data of Amblyopic Subjects

					Visual Acuity (logMAR)			Refraction				
Subject ID	Sex	Age (Y)	Type	Strabismus Angle	AE	FE	Severity of Amblyopia	AE	FE	Strabismus Distance (∆)	Stereopsis (log arc sec)	Dichoptic Motion Coherence (log Cum AUC)
1	F	15	Aniso	1	0.49	0.01	Moderate	+2.25+1.50 × 115	+0.50+0.75 × 080	Ortho	2	3.13
2	F	9	Aniso	0	0.337	0.07	Mild	Plano+4.00 × 095	+0.25+2.25 × 080	Ortho	2	3.13
3	F	12	Aniso	0	0.43	−0.06	Moderate	+5.50+2.00 × 080	+1.75+0.50 × 110	Ortho	2.30	3.23
4	M	10	Aniso	0	0.19	0.006	Moderate	+2.50+1.75 × 075	+0.75+0.50 × 095	Ortho	2	2.41
5	F	11	Aniso	0	0.36	−0.11	Moderate	+1.25+2.00 × 095	+0.50+0.25 × 085	Ortho	2.90	3.08
6	F	6	Aniso	0	0.31	−0.01	Moderate	+4.75+1 × 079	+2.50+1 × 084	Ortho	2	2.85
7	F	7	Aniso	0	0.77	0.06	Moderate	+4.50+0.50 × 085	+2.50+0.25 × 090	Ortho	2.90	3.12
8	M	7	Aniso	0	0.16	0.02	Moderate	+4.5+0.50 × 126	+1.75	Ortho	2	1.88
9	M	14	Aniso	0	0.11	0.09	Treated	+2.00+2.00 × 100	+3.75+1.50 × 065	Ortho	2	3.30
10	M	12	Aniso	0	0.31	0.01	Moderate	+4.50+1.25 × 073	+2.50+1.75 × 103	Ortho	2	3.00
11	F	14	Aniso	0	0.29	0.16	Mild	+6.00+1.25 × 088	+5.00+1.50 × 096	Ortho	2	2.36
12	F	18	Aniso	0	0.23	−0.01	Mild	−2.75+6.25 × 088	−0.75+1.50 × 082	Ortho	2.15	1.81
13	F	55	Mixed	20	0.52	0.13	Moderate	Plano+125 × 110	0.25	20 ET + 2 LH	3.85	3.61
14	M	9	Mixed	15.2	0.08	−0.02	Moderate	+2.00+1.50 × 105	+0.50+0.75 × 080	RX (T)14 LH (T)6	2	3.03
15	F	22	Mixed	1	0.01	−0.09	Treated	+6.50+0.75 × 030	+7.25	E (T')25	3.85	2.23
16	F	13	Mixed	1.7	0.24	−0.04	Moderate	−2.25+0.75 × 100	−3.50+1.50 × 080	XT (RHT?)	2	3.13
17	F	32	Mixed	8.3	0.40	0.04	Moderate	+7.00+0.50 × 147	+7.00+0.50 × 057	RHT8, RET2	3.54	2.87
18	M	10	Mixed	4	0.45	−0.02	Moderate	+1.25+3.75 × 095	+0.50+2.00 × 085	RET 4–6	2.30	3.28
19	M	13	Mixed	8.3	0.56	−0.10	Moderate	+1.75+2.50 × 110	+0.25+0.75 × 085	RET 2, right hypo 8	3.85	2.49
20	M	17	Mixed	7	0.33	−0.02	Moderate	1.00+1.75 × 090	1.25+4.50 × 095	LE (T) 6–8	3.85	3.22
21	F	30	Mixed	12.2	−0.01	−0.09	Treated	+2.25+0.75 × 136	−0.25+1.50 × 079	12 DVD, 2 E (T)	3.85	1.69
22	M	9	Mixed	1	0.31	0.08	Mild	+7.00+1.00 × 130	+7.5+0.5 × 71	Flick E	2	2.45
23	F	14	Mixed	4	0.18	−0.04	Treated	+5.00+1.75 × 075	+4.00+0.75 × 100	RET 4	2	2.02

DVD, dissociated vertical deviation; ET, esotropia; E(T), intermittent esotropia; E(T’), intermittent esotropia at near; Flick E, esodeviation <10∆; HT, hypertropia; Hypo 8, 8∆ hypotropia; HypoT, hypotropia (preceded by L for left or R for right); (), intermittent deviation; LH, left hypertropia; Ortho, orthotropia; RX, right exotropia; XT, exotropia.

Aniso indicates anisometropic amblyopia based on meeting at least one of the following criteria: ≥0.50 D difference between both eyes in spherical equivalent or ≥1.50 D difference between both eyes in astigmatism at any meridian. Strabismic indicates strabismic amblyopia based on meeting at least one of the following and criteria are not met for mixed amblyopia: (1) heterotropia at distance (with or without spectacles), (2) history of strabismus surgery, or (3) history of strabismus that has resolved with glasses and/or surgery. Mixed indicates mixed amblyopia based on meeting both of the following criteria: (1) criteria for strabismus (see above) and (2) ≥1.00 D difference between both eyes in spherical equivalent or ≥1.50 D difference between eyes in astigmatism in any meridian. Severity of amblyopia is classified into two groups: treated (for participants with treated amblyopia) and mild, moderate, and severe as per PEDIG studies.^50^ Absence of stereopsis (nil) is indicated by a logStereopsis value of 3.85 (represents 7000 arcsec).

### Measurements of Visual Functions

Monocular visual acuity was assessed at 3.1 meters in a dark room using Early Treatment of Diabetic Retinopathy Study (ETDRS) optotypes with crowding bars on a 32-inch Display++ LCD monitor (1920 × 1080 resolution, 120 Hz, 111-cd/m^2^ brightness; Cambridge Research Systems, Rochester, Kent, UK).^6^^,^^8^^,^^51^ Optotype sizes in arcminutes were adjusted using an adaptive staircase procedure with six reversals, and the logMAR acuity was calculated as the log of the threshold representing the arithmetic mean of the reversals. We evaluated interocular suppression using the dichoptic motion coherence test on the Display++ LCD monitor operating in interleaved stereo mode with alternating scan lines directed to each eye with polarized glasses in a dark room.^6^^,^^52^^–^^54^ Suppression was quantified by fitting a third-order polynomial to the log of the number of signal dots required at each noise contrast level. The log-transformed value of the cumulative area under this fitted curve (log CumAUC) served as the suppression index. Larger log CumAUC values indicated stronger suppression. Stereopsis was measured in arcseconds using the Titmus Stereoacuity Test.^6^^,^^7^ Participants with absent stereopsis were assigned a value of 7000 arcsec (3.85 log arcsec).

### Visual Search Task

**Figure 1.**
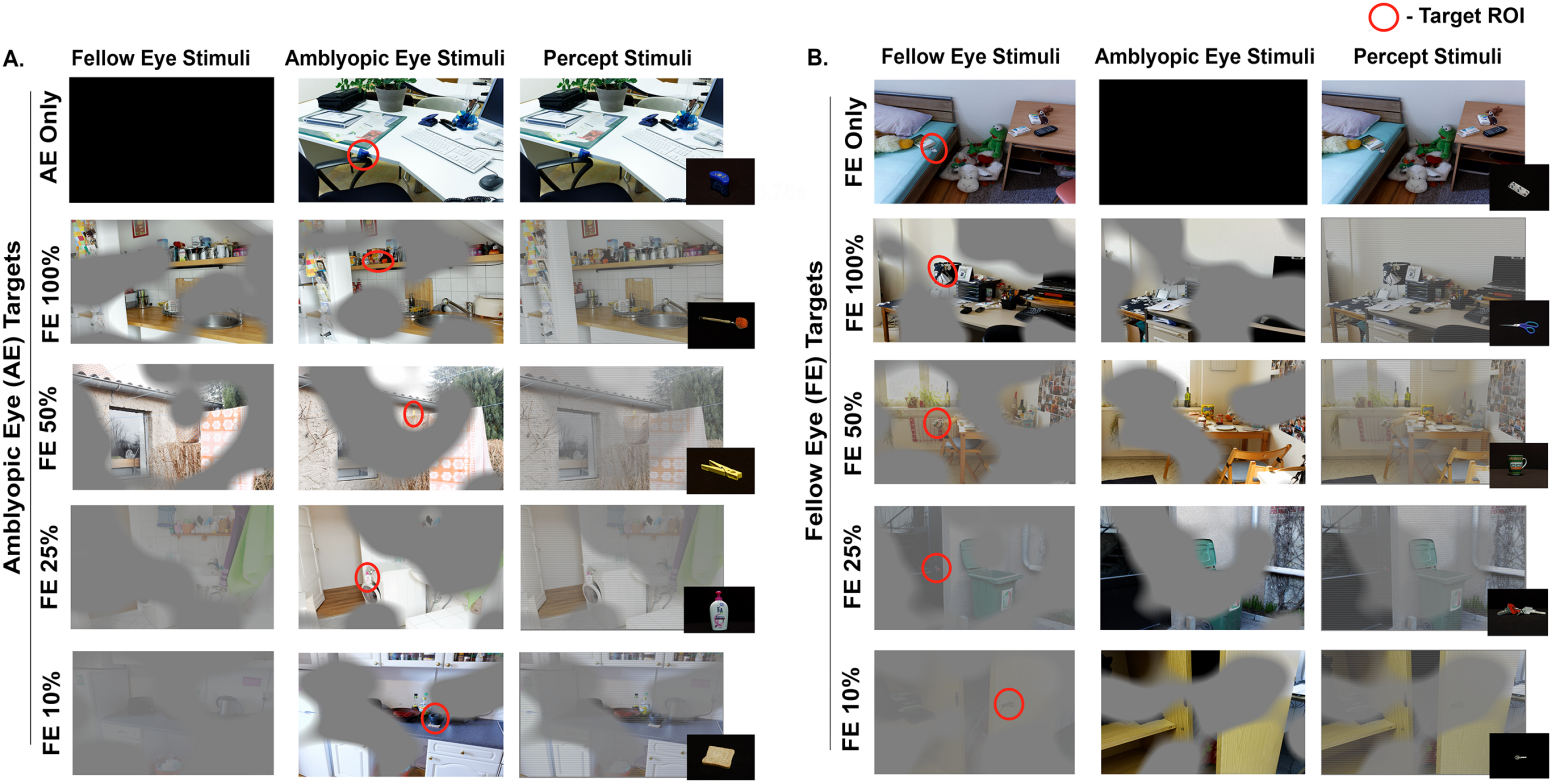
**Experimental paradigm for visual search task.** (**A**, **B**) Illustration of visual search tasks with targets in the AE region (**A**) or FE region (**B**). The *left* and *middle columns* show stimuli presented to FE and AE, respectively. The *right column* shows the combined percept of AE and FE stimuli. *Red circles* mark the ROI. *Rows* indicate monocular (AE only or FE only) and dichoptic viewing with FE contrast at 100%, 50%, 25%, and 10%. In dichoptic trials, participants combined AE and FE inputs to perceive the full image.

Trial images were presented dichoptically using the 32-inch Display++ LCD monitor (84-cm viewing distance) in a dark room. Experiments were created with Psykinematix (KyberVision, Miyagi, Japan).^34^^,^^52^ Subjects were first shown an image of a target object (Fig. 1) and given 10 seconds to locate and click on that target object within a complex visual scene. The click location and time were recorded. The 32 trials included eight monocular and 24 dichoptic trials. In the monocular trials, images were shown to either the AE (Fig. 1A) or FE only (Fig. 1B), four trials each.

In dichoptic viewing, irregularly shaped Gaussian-edged masks were generated using MATLAB (MathWorks, Natick, MA, USA) and applied to separate regions of the stimulus presented to each eye. The AE (or right eye for controls) viewed one masked region, and the FE (or left eye for controls) viewed the complementary inverse with overlapping transition zones that were visible to both eyes. This produced a percept that required binocular integration to reconstruct the full image, similar to the approach used in Luminopia (a U.S. Food and Drug Administration [FDA]–approved therapy).^25^^,^^34^^,^^47^^,^^52^^,^^55^^,^^56^ In the dichoptic trials, AE contrast was fixed at 100%, but FE contrast varied across four levels (100%, 50%, 25%, and 10%). Each contrast level included six trials: three with AE-visible targets (AE targets, Fig. 1A) and three with FE-visible targets (FE targets, Fig. 1B). Trial order was counterbalanced and randomized across viewing conditions and targets.

### Eye Movement Recording and Data Analysis

Monocular calibration and validation were performed prior to each session. Binocular eye positions were recorded using the EyeLink 1000 Plus (SR Research, Ottawa, ON, Canada) at a sampling rate of 500 Hz and spatial resolution of 0.01°. Eye velocity was computed by differentiating eye position signals, applying a Savitzky–Golay filter for smoothing, and was used for identifying and excluding blinks and partial blinks.^57^^,^^58^ Saccades and microsaccades were detected using the Engbert and Kliegl velocity-based algorithm.^59^ Microsaccades were defined as saccades with amplitudes ≤ 1°.^33^ We removed 20 ms of data from the start and end of each saccadic event to exclude acceleration and deceleration artifacts associated with rapid eye movements. We pooled together microsaccades, volitional saccades, and quick phases, based on their shared oculomotor dynamics, to compute frequencies, amplitudes, and intersaccadic intervals.^4^ Saccade onset and offset times were used to segment fixation periods. Within each fixation, composite drift velocities of each eye were computed.

For each trial, a ROI was defined in pixel coordinates to encompass the target stimulus along with a surrounding circular area subtending a visual angle of 1°. This ensured that the ROI captured both direct fixations on the target and those in its immediate vicinity. Gaze data from both eyes were analyzed to identify fixation events throughout each trial, including those that occurred within the predefined ROI. A fixation was considered to fall within the ROI only if its centroid landed inside the predefined target region, which consisted of the target stimulus plus a 1° visual-angle margin. To qualify as a “look,” a fixation within this ROI had to last at least 100 ms, consistent with established thresholds in other eye-tracking–based ROI studies.^60^^,^^61^ To quantify visual engagement, we measured the time elapsed from the onset of the image to the final fixation (look time) of the FE for FE-target trials and the AE for AE-target trials.^60^^,^^61^ This metric, referred to as “look behavior,” served as an index of visual attention directed toward the target throughout each trial.

### Perceptual Assessment and Response Categorization

To assess whether the target object within a trial was perceived, we recorded the presence or absence of a correct mouse click. The performance within each trial were categorized into one of three perceptual outcomes:

• *Look and see*—Fixation occurred within the ROI, and the participant made a correct mouse-click response indicating both visual attention and successful perception.
• *Look but failed to see*—The participant fixated within the ROI but either made an incorrect response (e.g., incorrect mouse click) or failed to respond (e.g., trial timeout), suggesting perceptual failure despite visual attention.
• *No look, no see*—No fixation occurred within the ROI, and the response was either incorrect or absent, indicating both attentional and perceptual failure.

We computed the action time (i.e., the interval from the final fixation within the ROI to the mouse click) for correct trials to evaluate whether the duration of action differed between amblyopic and control participants after the target was located.

We computed a metric reflective of accuracy of DiVS performance for AE and FE targets. For each participant, we plotted the percentage of correct trials—that is, look and see trials (*y*-axis) against FE contrast (*x*-axis). We calculated the area under the curve (AUC) of connected data points representing the accuracy-versus-contrast function using the piecewise cubic Hermite interpolating polynomial (PCHIP). This method was selected to ensure a shape-preserving fit that respects the local data range and avoids artificial oscillations (Runge's phenomenon) common in sparse datasets.^62^^,^^63^ We then computed the area under this curve and derived the log_10_ cumulative area under the curve (logAUC_DiVS) as a measure of DiVS performance for AE and FE targets separately, with lower values indicating greater difficulty identifying targets. A detailed schematic illustrating the calculation of the integral used to derive the logAUC_DiVS metric along with representative accuracy-versus-contrast functions from example study participants are presented in Supplementary Figure S1.

### Quantification of Temporal Control of Eye Deviation Using DBSCAN

To analyze eye alignment, horizontal and vertical eye position data were first smoothed using a moving average filter to remove fast eye movements and isolate fixational positions. The difference in eye position between the left and right eye was then computed, and composite eye alignment, defined in Equation 1a, was calculated for each trial and participant. To assess temporal control of alignment, eye deviation data were clustered using a two-dimensional (2D) DBSCAN algorithm,^64^^–^^66^ which identifies stable alignment periods based on data density. Parameters were tuned to exclude rapid transitions while avoiding excessive fragmentation. Clusters were classified by their mean position: Those within 3.5° horizontally and 2.0° vertically were labeled as well-aligned, and others were considered misaligned. To prevent oversegmentation, clusters with centroids within 1° horizontally and 0.5° vertically were merged into unified alignment states, applying the same criteria to both well-aligned and misaligned clusters. From the final clusters, a time-based weighted mean eye deviation was derived that quantifies the average magnitude of eye deviation clusters weighted by the proportion of time spent in each cluster using Equations 1b to 1d:

(1) $$\begin{array}{c} \mathrm{Composite}=\sqrt{\left(\mathrm{Horizonta}l^{2}+\;\mathrm{Vertica}l^{2}\right)}\; \end{array}$$

(2) $$\begin{array}{c} FT_{n}=T_{n}/T_{total}\\ {}[T_{total}=T_{1}+T_{2}] \end{array}$$

(3) $$\begin{array}{c} \mathrm{ClusterComposit}e_{n}=\mathrm{Composit}e_{n}*FT_{n} \end{array}$$

(4) $$\begin{array}{c} \mathrm{Mea}n_{Weighted}=\left(\sum_{}^{}ClusterComposite_{n}\right)/T_{total} \end{array}$$

A higher time-weighted mean value reflects a greater degree of sustained eye deviation over extended periods.

### Statistical Analysis

Group differences in age between control and amblyopic participants were evaluated using the Mann–Whitney *U* test. To examine differences in task performance, χ^2^ tests were used to compare the distribution of the three key perceptual outcomes between control and amblyopic participants. To assess group-level differences in the trial outcomes, three-way linear mixed-effects models were utilized with Bonferroni-corrected post hoc comparisons. The dependent variables included look time, action time, ocular motor measures (saccade frequency, microsaccade frequency, time-based weighted means of eye deviation, and composite drift velocity), and visual sensory measures (interocular suppression, AE visual acuity, and stereoacuity). The models incorporated one between-participants factor (group: control vs. amblyopic) and three within-participants factors: click response (correct vs. incorrect), viewing conditions (monocular, FE 100%, FE 50%, FE 25%, FE 10%), and targets (AE vs. FE targets) with age of participants as a covariate. We also explored within-group differences between click response across viewing conditions and targets using a separate three-way mixed-effects models with Bonferroni-adjusted pairwise comparisons. Normality of the data distributions was tested using the Kolmogorov–Smirnov test. Levene's test of equality of error variances and Mauchly's test of sphericity were applied to confirm assumptions of homogeneity of variance and sphericity, respectively. Pearson correlation analyses were conducted to examine the relationship between age and the accuracy of AE and FE target detection during the DiVS task, quantified as logAUC_DiVS. Additional Pearson correlations were performed to assess associations between detection accuracy (logAUC_DiVS) and measures of suppression, stereoacuity, and AE visual acuity deficits. Overall target detection accuracy was compared between control and amblyopic participants using independent samples *t*-tests. Assessment of within-session stability was performed via split-half reliability analyses, utilizing paired *t*-tests to compare performance across the first and second halves of each session. All statistical analyses were carried out using SPSS Statistics (IBM Corporation, Chicago, IL, USA) and MATLAB, with the significance threshold set at α = 0.05. To account for multiple comparisons, Bonferroni correction was applied.

## Results

There were no significant age differences between control (11.43 ± 5.83 years) and amblyopic (15.61 ± 10.83 years) participants (*P* > 0.05). We evaluated the accuracies of detecting AE and FE targets across controls and amblyopic participants. For AE targets, a statistically significant difference in overall performance was observed between controls (86.32% ± 9.10%) and amblyopic participants (69.79% ± 22.56%; *P* = 0.004), with controls demonstrating superior accuracy. To assess the stability of measurements within each session, we performed a split-half reliability analysis comparing accuracy in the first and second halves of the trials for both groups. No significant difference was found between the first half (90.35% ± 8.65%) and the second half (82.29% ± 16.01%) among control participants (*P* = 0.1). Similarly, no significant differences between the first half (71.88% ± 27.32%) and the second half (67.71% ± 22.51%) were seen among the amblyopic participants (*P* = 0.900).

For FE targets, there was no significant difference in overall performance between controls (52.98% ± 10.65%) and amblyopic participants (52.90% ± 15.20%; *P* = 0.987). Measurement stability was also confirmed for FE targets, with no significant differences between the first and second halves for either control participants (first half: 52.38% ± 14.41% vs. second half: 53.57% ± 14.88%; *P* = 0.831) or amblyopic participants (first half: 50.72% ± 21.01% vs. second half: 55.07% ± 15.44%; *P* = 0.428). These results confirm a substantial deficit in AE target detection among amblyopic participants compared with controls. However, accuracy scores alone cannot determine whether this reduced performance reflects an inability to locate the target (a failure to look) or an inability to consciously perceive the target despite adequate fixation (a failure to see). To disentangle these possibilities, we applied a look-versus-see framework and examined fixation patterns to quantify how often targets were fixated but nonetheless went unperceived.

### Fixation and Perceptual Behavior During AE Target Trials

**Figure 2.**
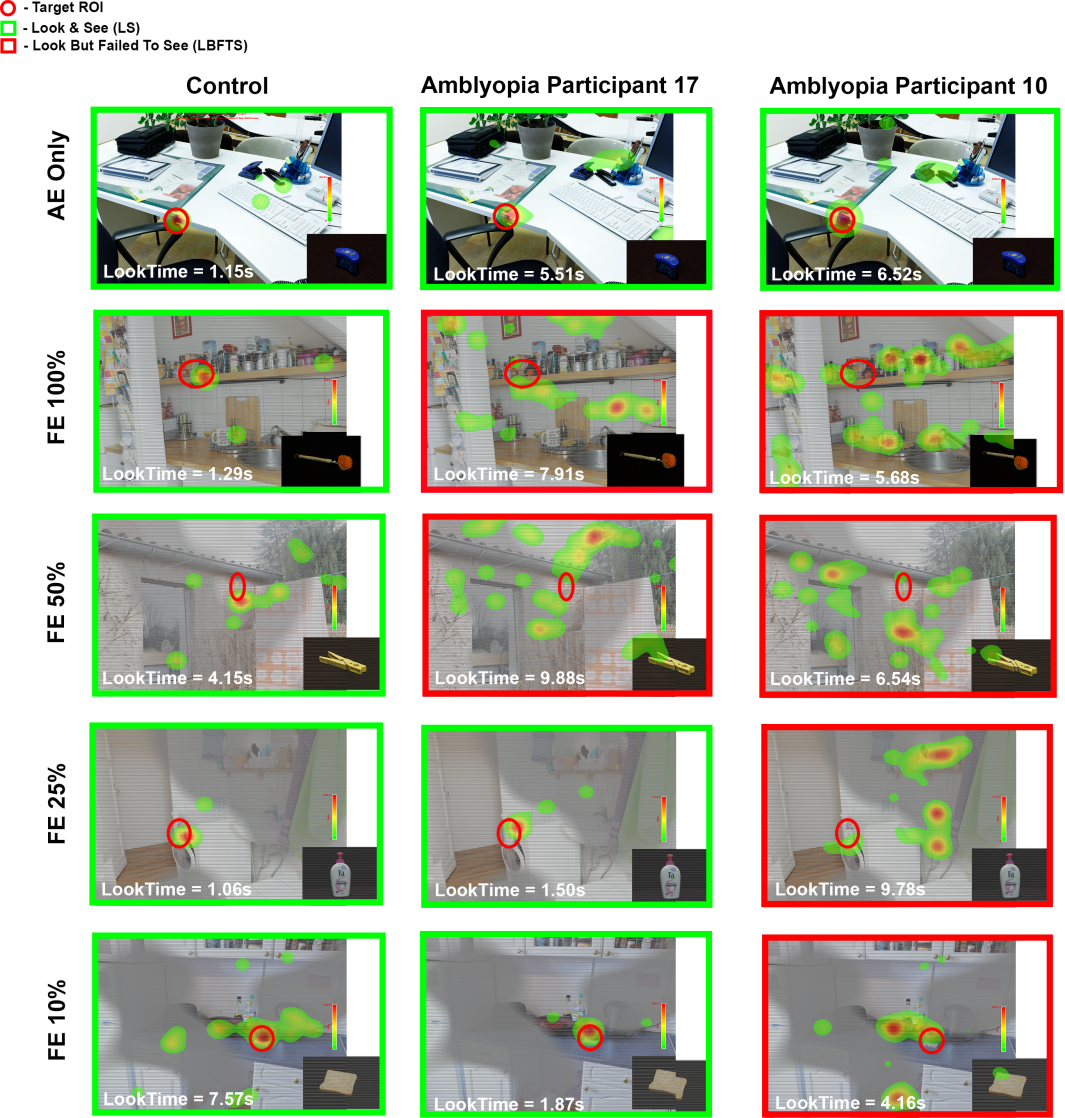
**Fixation heatmaps for amblyopic eye (AE) targets.** Representative control and two amblyopic participants (P17, P10) during AE target trials under monocular and dichoptic conditions. *Green* represents LS; *red*, LBFTS. *Red circles* mark target locations. Heatmaps show fixation density (warm = high). LookTime is the time from image onset to last fixation in ROI. NA indicates that the participant neither located nor identified the target.

Figure 2 illustrates fixation behavior with the look times across monocular and dichoptic viewing for a control participant and two individuals with amblyopia during AE target trials. The control participant consistently fixated within the ROI and correctly identified the target across all conditions, as shown in the fixation heatmaps (Fig. 2, panel 1). This pattern reflects robust binocular integration and perceptual success (look and see). Both amblyopic participants demonstrated perceptual success under monocular AE viewing (look and see), but the reaction times were increased compared to the control participant. Under dichoptic conditions, performance diverged between participants 17 and 10. Participant 17, who exhibited less suppression (logAUC = 2.87) (Fig. 2, panel 2), performed better than participant 10, who showed greater suppression (logAUC = 3) (Fig. 2, panel 3). Participant 17 fixated but failed to locate the AE target at FE 100% contrast and FE 50% (look but failed to see) but successfully identified the target (look and see) at lower FE contrasts (25% and 10%). This indicated an ability to overcome suppression with contrast rebalancing. In contrast, participant 10 consistently fixated within the ROI across all FE contrast levels but failed to identify the AE target, demonstrating persistent suppression despite accurate gaze allocation (look but failed to see).

### Fixation and Perceptual Behavior During FE Target Trials

**Figure 3.**
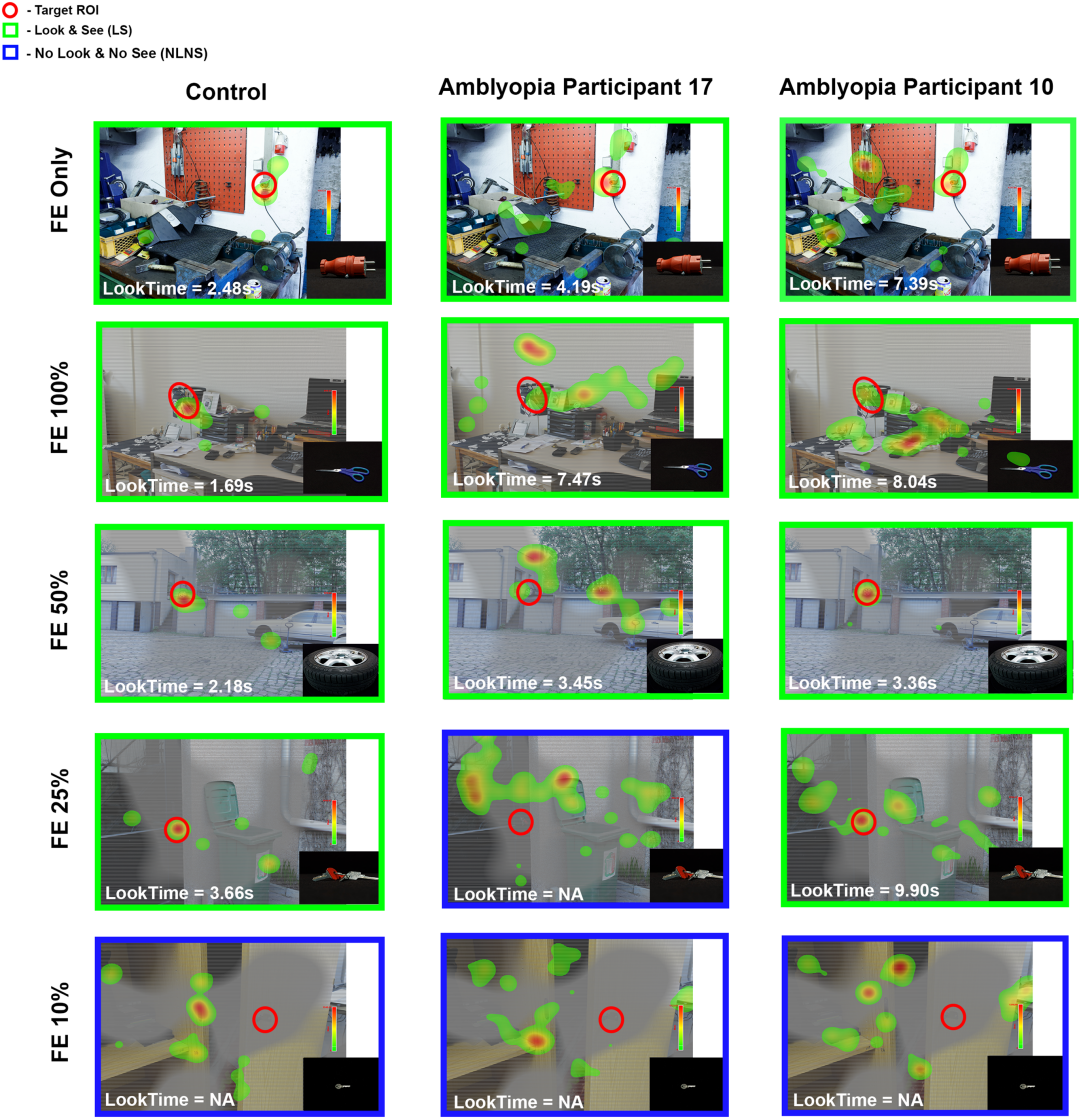
**Fixation heatmaps for fellow eye (FE) targets.** Representative control and two amblyopic participants during FE target trials. *Green* represents LS; *blue*, NLNS. *Red circles* mark target ROI locations; heatmaps show fixation density. LookTime is the time from image onset to last fixation in ROI. NA indicates that the participant neither located nor identified the target.

Figure 3 presents fixation behavior during FE target trials for the same participants as in Figure 2. All three participants successfully fixated within the ROI and identified the target (look and see) under monocular FE only and dichoptic viewing at 100% and 50% FE contrast, as shown by their fixation heatmaps. The control participant consistently demonstrated rapid and efficient target acquisition with shorter look times across all conditions compared to participants 17 and 10, with the slowest acquisition and longest look times for participant 10. At 25% FE contrast, both the control and participant 10 located and identified the target, whereas participant 17 failed to locate the target. At the lowest FE contrast level (10%), none of the participants was able to detect or identify the target, resulting in “no look, no see” trials.

### Group-Level Analysis of Perceptual Outcomes

**Figure 4.**
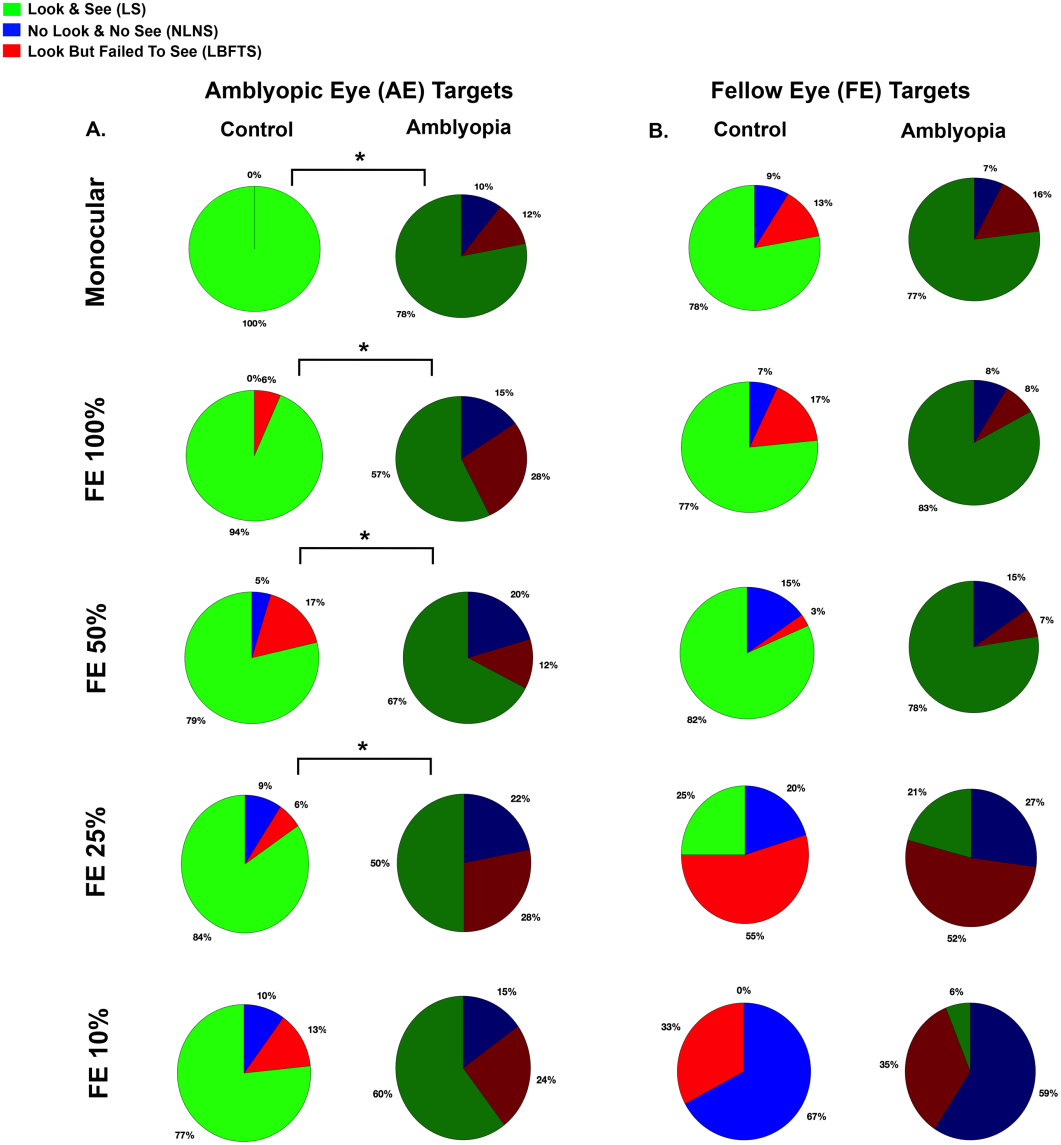
**Pie chart visualization of key perceptual outcomes.** (**A**) The pie charts show scenario distributions for controls versus amblyopia participants for AE targets; significant group differences occurred at AE only, FE 100%, FE 50%, and FE 25%. (**B**) Scenario distributions for the FE targets show that significant group differences occurred only at FE 100%. *Significant χ^2^ (Bonferroni corrected).

We used pie charts to examine group-level differences in how controls and amblyopic participants perceived AE and FE targets—look and see (LS), look but failed to see (LBFTS), and no look, no see (NLNS)—across the monocular and dichoptic viewing conditions. Figure 4A shows pie charts illustrating the perceptual outcomes for AE targets in control and amblyopic participants. Significant group differences were observed for AE only (χ^2^ = 22.059; *P* < 0.001), FE 100% (χ^2^ = 26.222; *P* < 0.001), FE 50% (χ^2^ = 8.344; *P* = 0.015), and FE 25% (χ^2^ = 20.157; *P* < 0.001). Controls had significantly higher proportion of LS trials than amblyopia participants for AE only (*P* < 0.001), FE 100% (*P* < 0.001), and FE 25% (*P* < 0.001). Amblyopia participants had a higher proportion of LBFTS trials for AE only (*P* < 0.001), FE 100% (*P* < 0.001), and FE 25% (*P* < 0.001). Amblyopia participants also had higher proportions of NLNS trials for AE only (*P* = 0.002), FE 100% (*P* = 0.001), FE 50% (*P* = 0.004), and FE 25% (*P* = 0.039). We further examined the relationship between age and the accuracy of detecting AE targets across varied FE contrasts using logAUC_DiVS. A significant inverse correlation was observed (*R* = −0.43; *P* = 0.008), indicating that older participants exhibited reduced ability to identify AE targets during the DiVS task. Figure 4B shows pie charts illustrating the perceptual outcomes for FE targets in control and amblyopic participants. We observed no significant group differences between control and amblyopic participants for FE targets under all viewing conditions. However, reducing FE contrast to 25% or below impaired stimulus detection in both groups, likely due to diminished FE stimulus salience at low contrasts. Moreover, no significant correlation was found between age and logAUC_DiVS for FE targets (*R* = 0.042; *P* = 0.411), indicating that FE target identification performance was independent of age.

**Figure 5.**
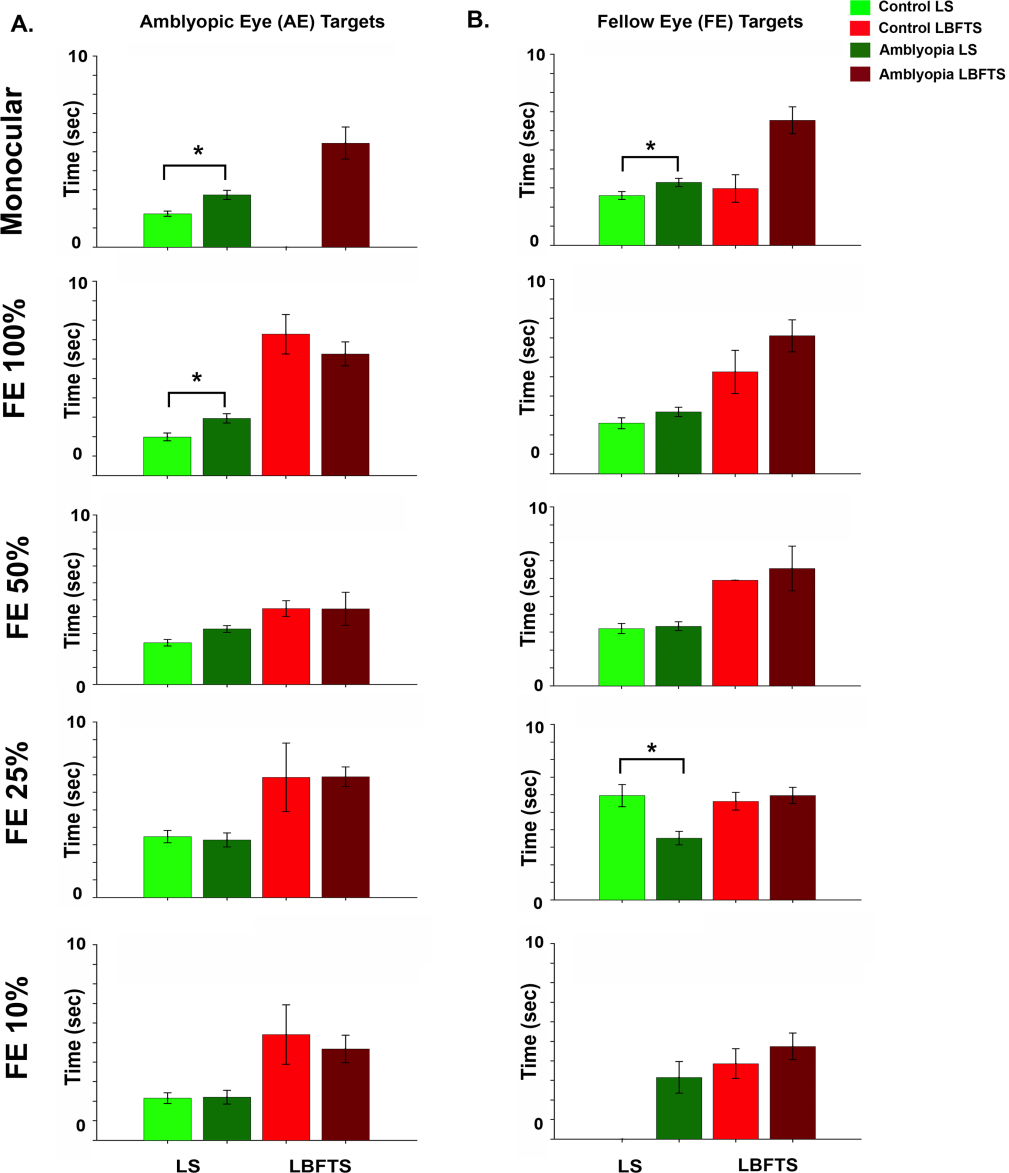
**Search efficiency between controls and amblyopia participants.** (**A**) Mean look times for LS and LBFTS trials for AE targets. Amblyopia participants were slower at AE only and FE 100% but matched controls at lower FE contrasts. Lowering FE contrast improved AE target detection and search efficiency. (**B**) For FE targets, the amblyopia group was faster at FE 25%, with no differences at other contrasts. Performance declined in both groups at FE 25% and especially FE 10%. *Significant χ^2^ (Bonferroni corrected).

Figure 5 illustrates look times for LS and LBFTS trials between controls and amblyopia participants across monocular and dichoptic viewing for AE and FE targets. Amblyopic participants showed altered visual search ability compared to controls for LS trials, with longer look times and significant difference across viewing conditions (*F* = 5.891; *P* < 0.001) and across targets (*F* = 5.734; *P* = 0.017) with no significant differences in age (*F* = 0.013; *P* = 0.909). There were also significant interactions between participant groups and viewing conditions (*F* = 4.012; *P* = 0.003) and between targets and participant groups (*F* = 4.145; *P* = 0.042). We found no significant differences in look times among viewing conditions (*F* = 1.234; *P* = 0.298), targets (*F* = 0.790; *P* = 0.375), participant groups (*F* = 0.083; *P* = 0.774), and age (*F* = 0.173; *P* = 0.678) for LBFTS trials. Figure 5A shows LS and LBFTS look times for AE targets across varying FE contrast levels. Amblyopia participants showed significantly longer look times at AE Only (P = 0.011) and FE 100% (*P* = 0.034), indicating delayed target acquisition. As FE contrast decreased, these delays diminished, with no significant differences at FE 50% (*P* = 0.152), FE 25% (*P* = 0.972), or FE 10% (*P* = 0.911). Together, these findings indicate that reducing FE contrast improves target identification and reduces look times in amblyopia. Figure 5B shows the LS and LBFTS look times for FE targets across varying FE contrast levels. Amblyopic participants exhibited significantly longer look times when the FE target was presented alone, FE only (*P* = 0.042), indicating increased difficulty in target acquisition. However, at 25% FE contrast, their fixation times were notably reduced (*P* = 0.008), suggesting improved target detection. Interestingly, at the lowest contrast level (FE 10%), a few amblyopic participants were still able to accurately detect the FE target, whereas none of the control participants succeeded in the task. Although reducing FE contrast to 25% or below significantly impairs FE stimulus detection, contrast rebalancing may still encourage amblyopic individuals to attend to both FE and AE stimuli, even when stimulus salience is low.

Time to action, measured from the final fixation within the ROI to the mouse click, did not differ significantly between age (*F* = 0.284; *P* = 0.594) and participant groups (*F* = 0.019; *P* = 0.892) for LS trials, indicating that amblyopic participants were as efficient as controls in responding once the target was located.

### Sensory Factors Impacting Perceptual Outcomes in Amblyopia

We assessed the influence of visual sensory factors—including interocular suppression, stereoacuity, and AE visual acuity—on visual search performance for AE and FE targets under dichoptic viewing by correlating these parameters with logAUC_DiVS. Greater interocular suppression and AE visual acuity deficits were significantly associated with poorer detection of AE targets (suppression: *r* = −0.46, *P* = 0.006; AE visual acuity: *r* = −0.47, *P* = 0.004), whereas stereoacuity showed no significant relationship (*r* = −0.01; *P* = 0.47). In contrast, FE target detection during DiVS was not correlated with suppression (*r* = −0.20; *P* = 0.14), stereoacuity (*r* = −0.13; *P* = 0.24), or AE visual acuity deficits (*r* = 0.07; *P* = 0.35). Bonferroni correction was applied to account for multiple comparisons, with statistical significance defined as *P* < 0.008.

**Figure 6.**
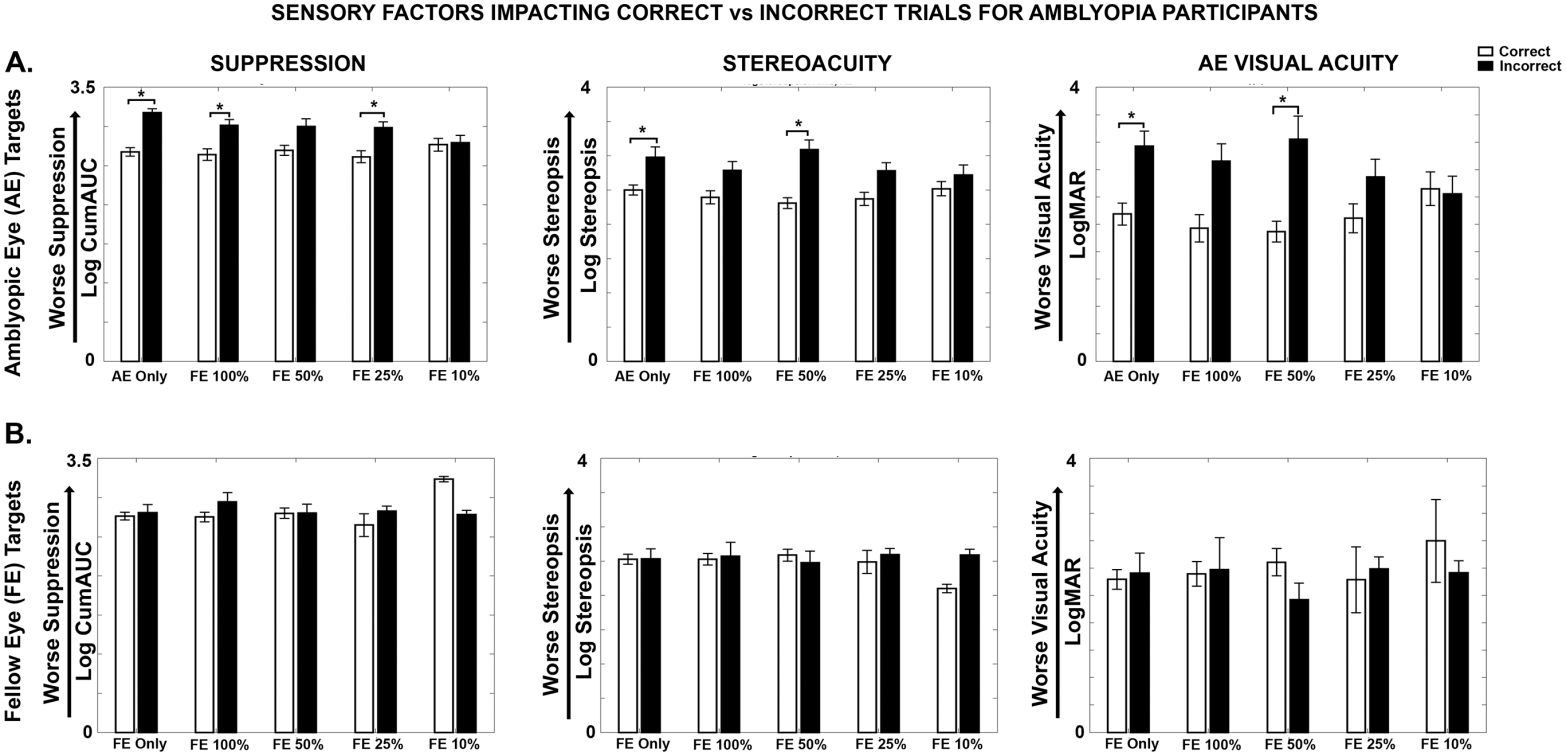
**Sensory factors for correct vs incorrect trials in amblyopia.** Comparison of suppression, stereoacuity, and AE acuity for correct (*white*) versus incorrect (*black*) responses. (**A**) AE targets. (**B**) FE targets. An asterisk (*) indicates a significant difference.

We further examined how visual sensory function deficits influenced performance for AE and FE targets across both monocular and dichoptic viewing conditions. For AE targets (Fig. 6A, left panel), higher interocular suppression was observed for incorrect (i.e., LBFTS and NLNS) trials compared to correct (LS) trials (*F* = 6.438; *P* = 0.011) and across age (*F* = 6.907; *P* = 0.009), with significant differences observed for AE only (*P* = 0.011), FE 100% (*P* = 0.029), and FE 25% (*P* = 0.030) viewing conditions. In contrast, no significant differences in suppression levels between correct and incorrect trials were observed for FE targets (Fig. 6B, left panel). For AE targets (Fig. 6A, middle panel), poorer stereoacuity was observed for incorrect compared to correct trials (F = 6.097; P = 0.014), with significant differences observed for AE only (*P* = 0.006) and FE 50% (*P* = 0.007) conditions. For FE targets (Fig. 6B, middle panel), there were no significant differences in stereoacuity between correct and incorrect trials. As illustrated in Figure 6A (right panel), worse AE visual acuity was seen for incorrect compared to correct trials (*F* = 4.304; *P* = 0.038), with significant differences noted under AE only (*P* = 0.008) and FE 50% (*P* = 0.002) conditions. For FE targets (Fig. 6B, right panel), no significant differences were observed in AE visual acuity between correct and incorrect trials.

### Motor Factors Impacting Perceptual Outcomes in Amblyopia

**Figure 7.**
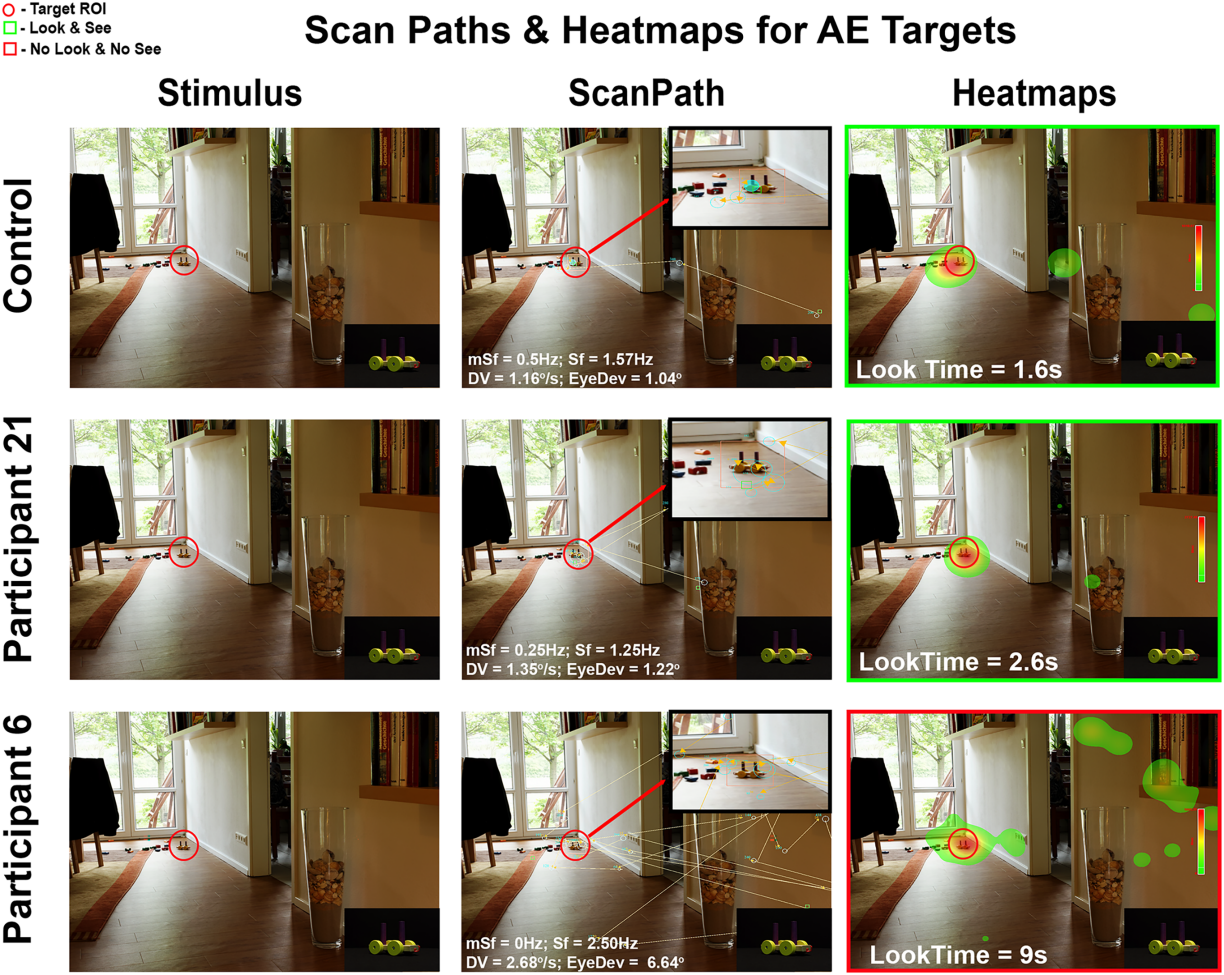
**Scan paths and fixation heatmaps.** Example from a representative control and two amblyopic participants (P21, P6) for the same AE target trial. (*Left*) Stimulus image with target ROI (*red circle*). (middle) Scan paths, with a *black box* showing a zoomed-in view of the ROI to highlight microsaccades (*yellow arrows*). Metrics for microsaccade frequency (mSf), saccade frequency (Sf), AE drift velocity (DV), and eye deviation (EyeDev) for each participant are provided. (*Right*) Fixation density heatmaps; the *green square* indicates correct identification and the blue square indicates NLNS. Look Time is the time from image onset to the last fixation in the ROI.

To investigate how motor factors are modulated by varying FE contrast levels, we first visualized search efficiency using scan paths and analyzed microsaccade and saccade frequencies, drift velocities, and eye deviation (Fig. 7, panel 2). Fixation heatmaps were used to visualize attention allocation and highlight differences between amblyopic and control participants (Fig. 7, panel 3). As illustrated in Figure 7, both the control participant and participant 21 (amblyopic) successfully located and accurately identified the target. This was confirmed by fixation heatmaps showing a high density of fixations within the ROI, and precise mouse click localization (LS is indicated by the green box in Fig. 7), suggesting effective attentional allocation and perceptual accuracy. Notably, participant 21 exhibited a longer look time than the control. Scan path analysis revealed concentrated microsaccades (small yellow arrows in the zoomed-in view in Fig. 7) within the ROI for both subjects. In contrast, participant 6 (amblyopic) also fixated within the ROI, as shown by the heatmaps with a prolonged look time of 9 seconds, but failed to identify the target (LBFTS is indicated by a red box in Fig. 7). Their scan path lacked microsaccades during the trial. Motor metric comparisons showed that participant 6 had greater eye deviation, higher AE drift velocity, and increased saccade frequency relative to both the control and participant 21. These findings suggest that elevated saccade frequency and eye deviation are linked to inefficient visual search and target detection failure, whereas the presence of microsaccades within the ROI correlates with successful identification.

**Figure 8.**
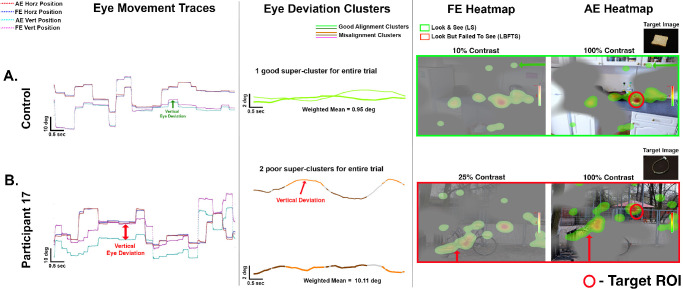
**Binocular alignment between control and persistently misaligned participant.** (*Left*) AE and FE horizontal/vertical eye position traces. (*Middle*) DBSCAN clusters. Green indicates good alignment; *orange*/*brown*/*pink*, misalignment. (*Right*) Fixation heatmaps for FE (low color intensity) and AE (high color intensity). (**A**) Control: stable alignment (weighted mean = 0.95°), single good cluster. *Green arrows* show overlapping eye positions in AE and FE heatmaps, validating good alignment with successful perception (LS). (**B**) P17 (amblyopia): persistent misalignment (10.11°), only misaligned clusters, non-overlapping fixations. *Red arrows* show a vertical shift in eye positions in the AE heatmap compared to the FE heatmap, confirming a vertical eye deviation with unsuccessful perception (LBFTS).

To assess the impact of binocular alignment stability on visual search performance, we analyzed eye movement traces, time-based weighted mean eye deviation clusters, and fixation heatmaps for the FE (low-intensity color) and AE (high-intensity color) within their respective stimulus regions (Fig. 8). The control participant (Fig. 8A, top panel) exhibited stable binocular alignment throughout the trial, as evidenced by tightly aligned AE and FE traces, overlapping fixation heatmaps of FE and AE in corresponding regions (Fig. 8A, green arrows), and a low weighted mean deviation of 0.95°. Cluster analysis revealed a single “good” super-cluster, indicating consistent alignment. This stability supported efficient and accurate target localization (LS is indicated by a green box in Fig. 8A), with fixations within the ROI. In contrast, participant 17 (amblyopic) (Fig. 8B, bottom panel) showed persistent binocular misalignment, reflected in a high weighted mean deviation of 10.11°. Cluster analysis identified two “poor” alignment groups. The fixation heatmaps revealed spatially distinct distributions for the FE and AE as indicated by the red arrows, suggesting that, although the FE actively engaged with its designated regions, the AE remained passively positioned in its region without effectively processing or perceiving the stimuli (LBFTS is indicated by a red box in Fig. 8B).

**Figure 9.**
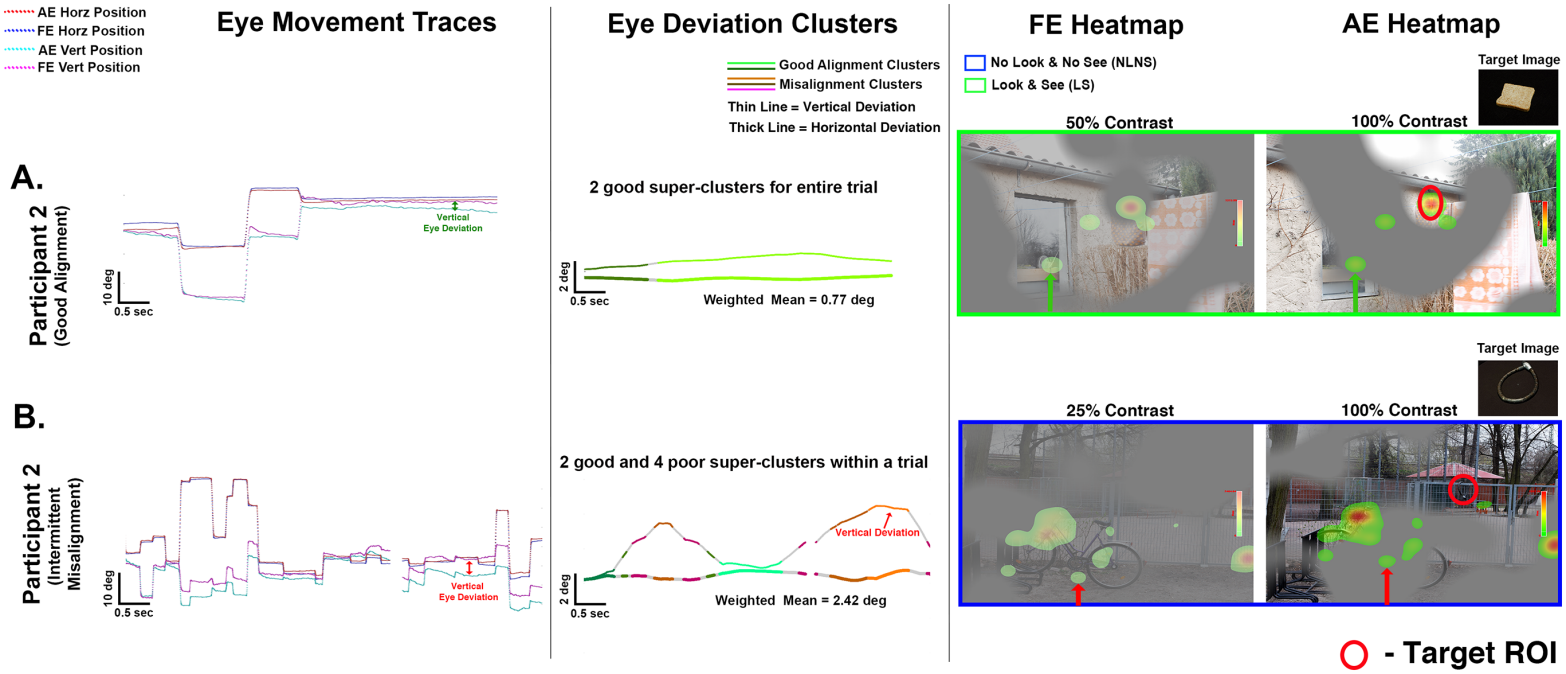
**Variable binocular alignment behavior within same participant.** (*Left*) AE and FE horizontal/vertical eye position traces. (*Middle*) DBSCAN clusters. Green indicates good alignment; *orange*/*brown*/*pink*, misalignment. (*Right*) Fixation heatmaps for FE (low color intensity) and AE (high color intensity). (**A**) P2 amblyopia: stable alignment (weighted mean = 0.77°), two good cluster leading to accurate localization of target as shown in heatmaps. *Green arrows* show overlapping eye positions in AE and FE heatmaps, validating good alignment with successful perception (LS). (**B**) P2 amblyopia: intermittent misalignment (weighted mean = 2.42°), mixed clusters, non-overlapping fixations. *Red arrows* show a vertical shift in eye positions in the AE heatmap compared to the FE heatmap, confirming a vertical eye deviation with unsuccessful perception (NLNS).

Figure 9 illustrates variability in ocular alignment across trials within the same amblyopic participant (participant 2). In Figure 9A, the participant demonstrated stable alignment, with tightly aligned AE and FE traces, a low weighted mean deviation of 0.77°, and two “good” super-clusters. The fixation heatmaps for both eyes overlapped in corresponding regions (Fig. 9A, green arrows), with fixations concentrated within the ROI, indicating that stable binocular alignment supported efficient and accurate target localization at 50% FE contrast. In contrast, Figure 9B shows intermittent strabismus in participant 2 while viewing a stimulus at 25% FE contrast. Cluster analysis revealed two “good” alignment clusters interspersed with four “poor” ones. The fixation heatmaps of FE and AE were non-overlapping, with the AE heatmap shifted upward and to the right, and a higher weighted mean deviation of 2.42°. The participant neither fixated on nor perceived the target, demonstrating that transient misalignments—even when interspersed with periods of good alignment—can disrupt perception in dichoptic environment despite lower FE contrast. These findings underscore the importance of stable binocular alignment for successful target identification.

**Figure 10.**
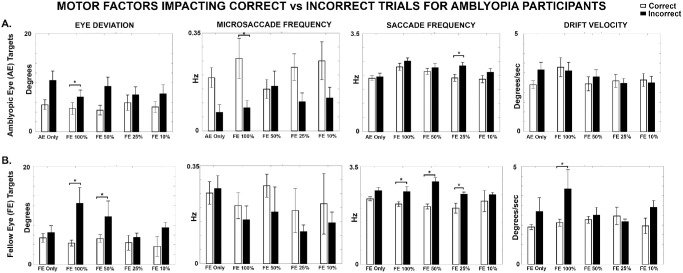
**Motor factors for correct versus incorrect trials in amblyopia.** Comparison of eye deviation, microsaccade frequency, saccade frequency, and drift velocity for correct (*white*) versus incorrect (*black*) trials for amblyopic participants. (**A**) AE targets. (**B**) FE targets. An asterisk (*) indicates a significant difference.

Based on the above insights, we examined the role of motor factors such as eye deviation, microsaccade and saccade frequencies, and drift velocities in search performance and evaluated how they are modulated by varying FE contrast levels as shown in Figure 10.

#### Eye Deviation

The time-based weighted means of eye deviation were generally higher on incorrect trials compared to correct trials (*F* = 12.721; *P* < 0.001), likely contributing to inefficient target detection. For AE targets, significant differences were found under FE 100% contrast (*P* = 0.048). For FE targets, a similar pattern was observed, with significantly greater eye deviation on incorrect trials at FE 100% contrast (*P* = 0.012) and FE 50% contrast (*P* = 0.032). These results were consistent with our individual participant-level observations that increased eye deviation is linked to unstable alignment, which results in impaired target acquisition during visual search.

#### Microsaccade Frequency

Microsaccade frequencies were generally higher during correct trials compared to incorrect trials (*F* = 8.308; *P* = 0.004) with significant differences noted for AE targets at 100% FE contrast (*P* = 0.015), indicating that increased microsaccades are associated with successful perception. No significant differences were found across age (*F* = 0.001; *P* = 0.974) and viewing conditions (*F* = 2.026, *P* = 0.155) for FE targets, although the trend of higher microsaccade frequency on correct trials was maintained.

#### Saccade Frequency

We observed that saccade frequencies were generally higher during incorrect trials compared to correct trials, potentially reflecting inefficient scanning strategies. We also found statistical significance between click responses (*F* = 21.616; *P* < 0.001), for age (*F* = 42.426; *P* < 0.001), and between targets (*F* = 19.073; *P* < 0.001). For AE targets (Fig. 10), a significant difference was observed at 25% FE contrast (*P* = 0.038). A similar pattern was seen for FE targets, with significantly higher saccade frequencies on incorrect trials at FE 100% (*P* = 0.033), FE 50% (*P* < 0.001), and FE 25% (*P* = 0.021) FE contrast levels.

#### Drift Velocity

We computed composite drift velocities of the AE for AE targets and FE for FE targets and found a significant overall difference between click responses (*F* = 6.200; *P* = 0.013) for amblyopia participants. However, although drift velocities were generally higher during incorrect trials, there were no significant differences across viewing conditions (*F* = 2.040; *P* = 0.087), age (*F* = 0.555; *P* = 456), or targets (*F* = 0.002; *P* = 0.969). We found significantly increased drift velocity only for the FE target at FE 100% (*P* < 0.001), suggesting that contrast reduction did not significantly influence drift velocities during visual search.

## Discussion

Visual search tasks require both adequate resolution and effective visual attention, feature binding, and executive function.^11^^,^^36^ Previous studies have shown that amblyopia impairs performance on such tasks, leading to slower visual processing and reading speeds, as well as suboptimal visual search strategies as quantified with eye tracking.^6^^,^^14^^,^^26^^,^^33^^,^^36^ This is the first study, to our knowledge, that has combined DiVS with high-resolution eye tracking to dissociate the sensory and motor contributions to perceptual deficits in amblyopia. We observed consistent difficulties in identifying AE targets among amblyopic participants, whereas performance differences for FE targets were comparatively minimal between amblyopic and control groups. Our findings confirm that visual search performance is significantly impaired in amblyopic participants during not only monocular but also dichoptic viewing, especially when the FE views stimuli at full contrast with an AE target. We also found that systematic reduction of FE contrast significantly improved AE target detection, confirming that contrast rebalancing facilitates both attentional allocation and conscious perception by overcoming suppression. This aligns with the principles of dichoptic therapy,^25^^,^^28^^,^^55^ and our results provide empirical evidence that such a strategy can improve functional vision. However, the benefits are not uniform. Participants with more severe deficits, such as greater AE visual acuity deficit, stronger suppression, and greater stereoacuity loss, and of an older age still struggled to perceive targets, even with lower FE contrast conditions at 10%. This suggests that, although contrast modulation can mitigate suppression, its efficacy is limited by the underlying severity of the visual deficits. We observed impaired perception of FE targets when FE contrast was reduced below 25%, which indicates that there is a critical lower bound for effective contrast modulation and binocular integration, emphasizing the importance of optimizing a balanced signal rather than simply minimizing FE input. We found no group differences in action times, which suggests that the primary limitations in amblyopia occur early in the search sequence, during attentional allocation and target detection, rather than during the final motor execution. This points to deficits in higher-order visual processing, such as feature binding and executive function.^11^^,^^32^^,^^37^^,^^67^^,^^68^

In addition to sensory factors, our findings highlight a strong relationship between oculomotor factors and visual search performance. To assess variability in eye deviation, we applied DBSCAN—a clustering algorithm well-suited for this task. DBSCAN identifies dense clusters that reflect sustained periods of stable eye alignment while effectively filtering out sparse, transitional points and noise, without requiring prior knowledge of the number of clusters. This approach enabled us to detect intermittent misalignment patterns throughout the viewing period. Time-weighted clustering of eye deviation revealed that stable binocular alignment is essential for accurate target localization and perception. In contrast, participants with intermittent or persistent misalignment exhibited poor perceptual outcomes. Our study provides the first cross-sectional evidence, based on objective eye-tracking measurements, demonstrating that greater ocular deviation is associated with poorer visual search performance in a dichoptic environment. These findings carry clinical relevance, as recent studies have begun evaluating dichoptic movie-based interventions aimed at reducing suppression and enhancing stereoacuity in individuals with strabismus without amblyopia, including those with intermittent exotropia.^69^^,^^70^ Taken together, our results highlight the importance of binocular alignment in dichoptic viewing and suggest that future randomized controlled trials should incorporate stratification or more homogeneous subgroup definitions to better isolate alignment-related mechanisms. We also observed that higher saccade frequencies were consistently associated with incorrect trials, suggesting that erratic or inefficient scanning strategies are associated with perceptual failures. Conversely, microsaccade frequencies were elevated in correct trials, indicating that these subtle eye movements may enhance fine-grained spatial attention and perceptual encoding as shown in previous studies.^45^^,^^46^^,^^71^

In the current study, in addition to traditional eye-tracking metrics, we also introduced a “look” versus “see” framework to better capture perceptual failures during visual tasks. This approach helps differentiate between two types of errors: perceptual failures, where the eyes never fixate on the relevant area (no look, no see), and post-attention failures, where the eyes do look at the region of interest, but the information is not processed correctly, likely due to persistent suppression (look but failed to see).^39^^,^^40^^,^^42^ This distinction highlights how suppression can play a dominant role in perception, even when eye movements appear appropriate. Our findings establish a strong connection between oculomotor behavior and perceptual success, demonstrating that both sensory and motor deficits contribute to impaired performance in DiVS tasks. A previous finding that performance can be improved in older children and adults supports the concept of neural plasticity beyond the traditional critical period.^20^^,^^22^^,^^28^^,^^31^^,^^49^^,^^72^ However, there is also evidence that neural plasticity may diminish with increasing age. Our results show that older participants experienced greater difficulty in correctly identifying AE targets, even though age did not influence look duration or time to action when the target had been located. This pattern suggests that age-related reductions in plasticity could potentially limit perceptual performance. Visual search tasks, when paired with eye-tracking analysis, offer a promising avenue for optimizing dichoptic stimuli and identifying biomarkers associated with perceptual performance. This approach offers a sensitive and objective tool for assessing functional vision in subjects undergoing dichoptic therapies.

In summary, our findings underscore the value of integrating DiVS with eye-tracking as a dual-purpose tool for amblyopia treatment. This combined approach enables precise, trial-level analysis of sensory–motor interactions and supports the determination of optimal contrast settings for personalized therapy—particularly for individuals with residual deficits or limited responsiveness to conventional methods. Given that our participants were 6 years and older, future research should focus on developing age-appropriate, pediatric-friendly stimuli and more engaging dichoptic tasks to effectively assess perceptual outcomes in younger children with age-matched controls. Current FDA-approved therapies typically employ a fixed, low FE contrast of approximately 15%. In contrast, our study provides quantitative evidence demonstrating systematic changes in perceptual performance across four distinct FE contrast levels using dichoptic stimuli with masking. These findings highlight the importance of individualized therapeutic stimulus design rather than a one-size-fits-all approach. Future studies could extend this framework by incorporating a broader range of FE contrast levels to more precisely characterize optimal, subject-specific contrast settings. Incorporating these elements, along with passive free-viewing paradigms and gaze-contingent tracking for therapeutic stimulus delivery, may enhance engagement and improve treatment outcomes. Future longitudinal studies with larger cohorts are essential to validate this approach, including follow-up measurement sessions with direct comparisons of results across time points to further establish test–retest reliability, monitor treatment-related changes over time, and refine adaptive contrast modulation strategies using real-time eye-tracking during therapy.

## Supplementary Material

Supplement 1
