# Supplementary material for: “Look Versus See”: Does Varying Fellow Eye Contrast Affect Perception of the Amblyopic Eye?
Source: Invest Ophthalmol Vis Sci. 2026 Apr 3;67(4):6. doi: 10.1167/iovs.67.4.6 (PMC13060727; doi:10.1167/iovs.67.4.6)
Supplement: Supplement 1 [file iovs-67-4-6_s001.pdf]

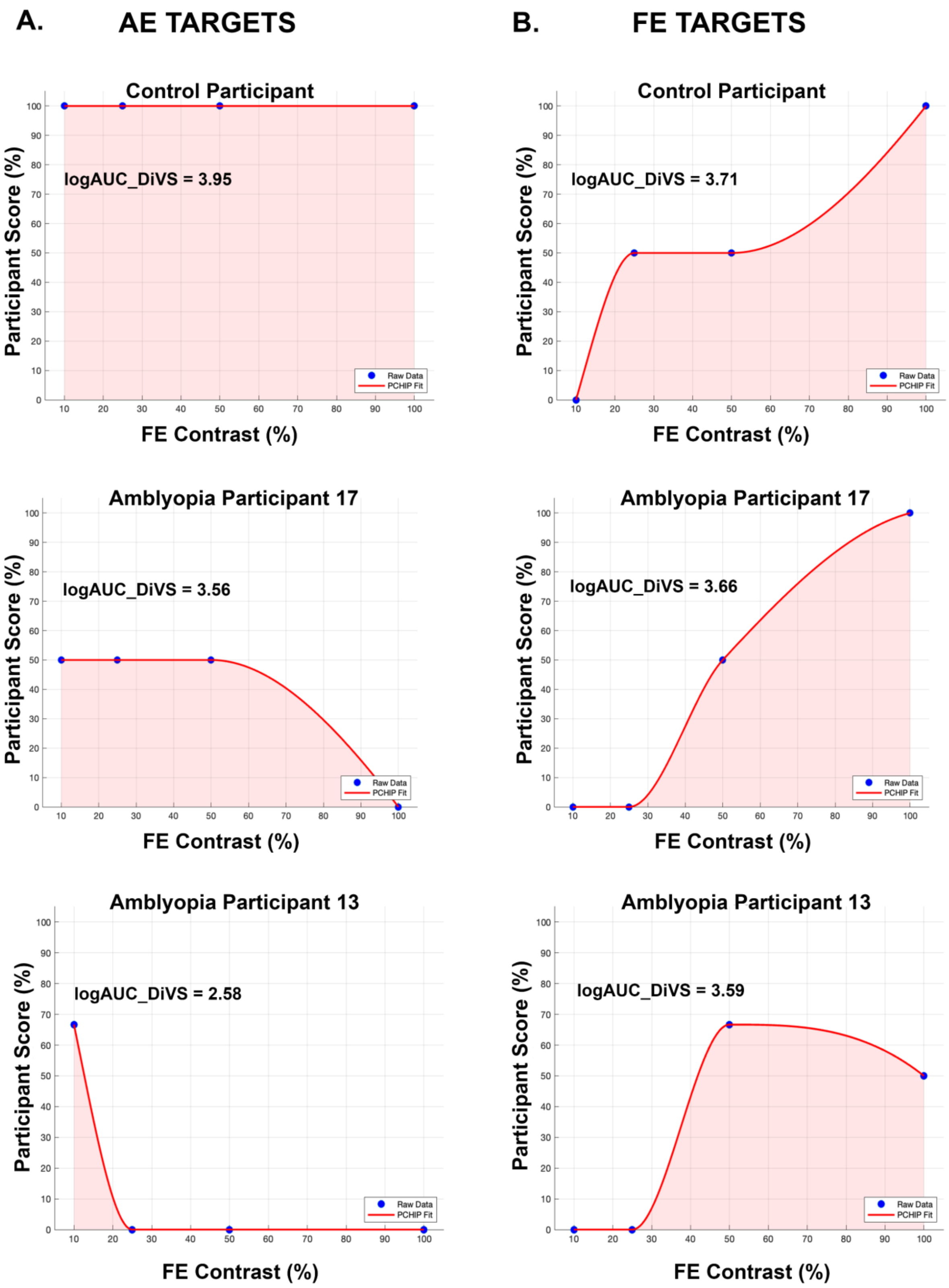

Supplementary Figure S1. Derivation of the logAUC_DiVS metric. Representative data showing accuracy (S) as a function of FE contrast (c) for amblyopic-eye (AE; A) and fellow-eye

(FE; B) targets in one control and two amblyopic participants. Circles denote raw accuracy at tested contrast levels, and solid curves show the Piecewise Cubic Hermite Interpolating Polynomial (PCHIP) fit used to model psychometric function of the performance across FE contrast. The shaded area represents the total Area Under the Curve (AUC) of the interpolated accuracy function, computed as the definite integral of the interpolated accuracy function:

$$\text{AUC} = \int_{c_{\min}}^{c_{\max}} S(c)$$

This value is log-transformed (log10AUC) to generate the final logAUC_DiVS metric which provides the measure of functional vision across the dynamic range of contrast sensitivity.

We would like to emphasize that logAUC_DiVS captures behavioral performance in the visual search task, whereas logAUC reflects the magnitude of sensory interocular suppression measured with dichoptic motion coherence. Thus, higher logAUC_DiVS denotes better performance, while lower logAUC denotes less interocular suppression.

Supplementary Figure Explanation:

AE Target Detection. (A): The logAUC_DiVS metric for AE targets captured the gradual *release of interocular suppression* as fellow-eye (FE) contrast was reduced.

- **Control Participant (logAUC = 2.13):** The control participant correctly identified all AE targets across all FE contrast levels. This performance resulted in a maximal logAUC_DiVS of **3.95**, reflecting the absence of AE suppression.
- **Participant 17** (**Less Suppression; logAUC = 2.87**): This participant, who exhibited moderate suppression as quantified using the dichoptic motion-coherence threshold and logMAR visual acuity of AE = 0.40, failed to identify AE targets at FE 100%, but performance improved to 50% accuracy at FE 50% and remained stable at lower FE contrasts, producing a logAUC_DiVS of 3.56.
- **Participant 13 (Deeper Suppression: logAUC = 3.61):** In contrast, Participant 13 who showed deeper suppression with greater visual acuity deficit of the amblyopic eye logMAR visual acuity of AE = 0.52), was unable to detect AE targets at FE 100%, 50%, or 25%, with only partial recovery at the lowest FE contrast, yielding a markedly reduced logAUC_DiVS (2.58). This delayed and steep recovery resulted in a substantially reduced area under the curve during dichoptic visual search of AE targets, reflecting greater sensory impairment relative to Participant 17.

FE Target Detection (B): For FE targets, all participants showed improved accuracy at higher FE contrasts.

- **Control Participant:** The control participant responded correctly on all FE 100% trials, with declining accuracy at lower FE contrasts. This yielded a logAUC_DiVS of 3.71.

- **Participant 17:** Participant 17 showed similar trends, achieving 100% accuracy at FE 100% and 50% accuracy at FE 50%, but failing to detect targets at FE 25% and FE 10%, resulting in a logAUC_DiVS of 3.66.
- **Participant 13:** Participant 13 showed lower overall accuracies than the other two subjects, achieving 50% accuracy at FE 100% and 66% at FE 50%, followed by complete failure at FE 25% and FE 10%. This pattern resulted in a slightly lower logAUC_DiVS value of 3.59.
